# Comparative accuracy of risk prediction models for mortality in acute coronary syndrome: A protocol for systematic review and meta analysis

**DOI:** 10.1097/MD.0000000000047675

**Published:** 2026-02-28

**Authors:** Yike Wang, Zhimei Chen, Jiantong Shen, Jianping Song, Meijuan Lan

**Affiliations:** a Department of Nursing, The Second Affiliated Hospital of Zhejiang University School of Medicine, Hangzhou, China; b Department of Cardiovascular Surgery, The Second Affiliated Hospital of Zhejiang University School of Medicine, Hangzhou, China; c Department of Nursing, The Second Affiliated Hospital of Guizhou University of Chinese Medicine, Guizhou, China; d School of Medicine, Huzhou University, Huzhou, Zhejiang, China; e Department of Medical, Huzhou Key Laboratory of Precise Prevention and Control of Major Chronic Diseases, Huzhou University, Huzhou, China.

**Keywords:** acute coronary syndrome, comparative accuracy evaluation, death risk prediction model, systematic review

## Abstract

**Background:**

The accuracy of different risk prediction models must be directly compared using research evidence from each model. This study systematically collected, evaluated and synthesized comparative accuracy data of mortality risk models for acute coronary syndrome (ACS) patients to compare their performance.

**Methods:**

An evidence-based approach was used to investigate ACS mortality risk prediction models. First, we searched multiple databases from 2009 to 2024, to identify multivariate predictive models for predicting ACS mortality risk. Included studies were screened, quality-assessed, and data extracted. PROBAST evaluated the risk of bias; heterogeneity was analyzed via MetaDiSc1.4 (*I*^2^ statistic). Data analysis used RevMan5.3 and MetaDiSc1.4. Sensitivity (SEN), specificity (SPE), positive/negative likelihood ratios (LR+/LR−), and area under the curve (AUC) of models were calculated for comparison.

**Results:**

A total of 8277 documents were retrieved, and 6 studies were finally included, involving 5 risk prediction models, a total of 24,911 patients with ACS, including 18,443 males (74.04%) and 6468 females (25.96%), with 1637 deaths. The SEN of the global registry of acute coronary events (GRACE) model was 0.78, SPE was 0.76, and AUC was 0.86; the SEN of the thrombolysis in myocardial infarction model was 0.51, SPE was 0.81, and AUC was 0.64; the SEN of the rapid emergency medicine score (REMS) model was 0.78, SPE was 0.46, and AUC was 0.41. The Acute physiology and chronic health evaluation II and REMS2 were reported separately due to non-combinable effect sizes, with SEN 0.77 to 0.95, SPE 0.22 to 0.99, and AUC 0.71–0.92. All 6 studies compared model accuracy. Pooled evidence indicated GRACE (AUC = 0.79) outperformed thrombolysis in myocardial infarction (0.59) and REMS (0.41); APACHE II (0.82) outperformed REMS (0.61) but was slightly inferior to GRACE (0.86).

**Conclusion:**

The GRACE risk prediction model is highly accurate and includes comprehensive clinical research data. It allows medical staff to accurately assess the death risk of ACS patients and effectively reduce their mortality. Therefore, the study suggests that clinical nursing staff use the GRACE risk prediction model to assess the risk of death in patients with ACS.

## 1. Introduction

Cardiovascular disease (CVD) is the leading cause of death and morbidity worldwide; it consumes enormous medical resources, imposes a growing medical economic burden, and has evolved into a serious public health issue.^[1-3]^ In China, CVD now has the highest mortality rate of any disease, accounting for 2 out of every five deaths.^[4]^ Acute coronary syndrome (ACS) is frequently the first clinical sign of CVD. ACS is a series of clinical syndromes caused by plaque fragmentation and damage to the vascular endothelium after coronary artery atherosclerosis, resulting in the accumulation of inflammatory factors and platelets forming a thrombosis, resulting in vascular lumen stenosis or even complete obstruction, and finally resulting in insufficient blood and oxygen supply to the myocardium.^[5,6]^ In 2019, there were an estimated 5.8 million new cases of ischemic heart disease in the 57 European Society of Cardiology member countries.^[2]^ The median age-standardized incidence estimate per 1,00,000 people was 293.3 (interquartile range 195.8–529.5).^[1]^ ACS develops rapidly, the prognosis is poor, and the mortality rate is high, making it one of the leading causes of death in European Society of Cardiology member countries.^[7,8]^ Studies^[9,10]^ have shown that using risk prediction models to estimate the risk of death in ACS patients can effectively screen out high-risk patients. This has important implications for selecting early intervention and optimal medical therapy for secondary prevention, potentially resulting in fewer complications, deaths, and waste of medical resources. One study^[4]^ showed that the global registry of acute coronary events (GRACE) risk prediction model is accurate in estimating the death risk of ACS patients, and the thrombolysis in myocardial infarction (TIMI) risk prediction model is similar in accuracy to the rapid emergency medicine score (REMS) risk prediction model. However, there is no systematic review of the accuracy and comparative accuracy of the predication of the risk of death for ACS patients. It is impossible to make evidence-based recommendations for existing risk prediction models, which affects medical staff in choosing an accurate and appropriate risk prediction model to evaluate ACS patients. This results in an unmet need for individualized ACS patient management strategies.^[11-13]^ Therefore, a scientific evaluation of ACS risk prediction models can help clinical nurses in screening out accurate and effective models, which can improve ACS patient care in ACS and reduce the risk of mortality.

## 2. Method

### 2.1. Search strategy

This study systematically searched English databases (Cochrane Library, MEDLINE, EMBASE, PubMed, Web of Science) and Chinese databases (WanFang Data Knowledge Service Platform, VIP, CNKI, and the China Biomedical Literature Database). Studies that used machine learning models were excluded because of potential heterogeneity in certain domains across the reviewed studies, which could prevent us from conducting a meta-analysis. The search period was between 2009 and 2024. A joint search was conducted based on the following subject words and free words: predict model/predict instrument/predict score/predict index/prognose model/prognose instrument/prognose score/prognose index/risk model/risk instrument/risk score/risk index/risk assessment model/risk assessment instrument/risk assessment score/acute coronary syndrome/coronary disease/coronary heart disease/myocardial infarction/myocardial infarct*/myocardial ischemia/angina/angina pectoris/angina pectoris/cardiac sudden death/sudden cardiac arrest/stenocardia*.

After computer retrieval, the references in the included literature were manually screened to identify studies on death risk prediction models for ACS patients. The original articles describing the development of various models were manually searched, and references to the included literature were reviewed to supplement the relevant literature. Supplemental Digital Content Files 1 to 4, Supplemental Digital Content, https://links.lww.com/MD/R372.

The study protocol followed the Declaration of Helsinki’s ethical guidelines. The Second Affiliated Hospital of Zhejiang University School of Medicine approved the study.

### 2.2. Inclusion and exclusion criteria of references

#### 2.2.1. Literature inclusion criteria

- The subjects were adult ACS patients; the content involved a comparative study of at least 2 risk prediction models for the prediction of the risk of death of ACS patients; the accuracy comparison data of the risk prediction model was used as the outcome measure.
- The literature included relevant outcome data such as sensitivity (SEN), specificity (SPE), relative risk (RR), comprehensive receiver operating characteristic (ROC), area under the curve (AUC), positive likelihood ratio (LR+), and negative likelihood ratio (LR−).

#### 2.2.2. Literature exclusion criteria

- The studies with only 1 risk prediction model’s development for ACS patients, without comparative analysis should be excluded.
- The literature content involved a comparative study of risk prediction models for intervention effects or other prognoses (e.g., bleeding, rehospitalization rate) of ACS patients.
- The research data was incomplete; statistical analysis was incorrect; unavailability of full text, and abstracts provided insufficient information.
- Machine learning models.

### 2.3. Ethical and dissemination

The protocol of the review does not require ethical approval because it does not involve humans. This article will be published in a peer-reviewed journal and presented at relevant conferences.

### 2.4. Literature selection and data extraction and evaluation

Two reviewers (WYK and CZM) independently screened the article titles and abstracts. Differences were resolved through multiple screenings and negotiations. Following consensus, the full-text articles were retrieved, and the data was extracted by 2 independent reviewers (WYK and CZM). When in doubt, a third (SJT) or fourth (SJP) researcher joined the discussion to reach a consensus. The extracted contents were: general information; general study characteristics; and research results: calibration and discrimination between risk prediction models.

### 2.5. Bias risk assessment

The Prediction model Risk Of Bias Assessment Tool (PROBAST) evaluation tool assessed the risk of bias in the literature.^[14]^ The specific PROBAST evaluation table can be found in the Supplemental Digital Content Files 2, Supplemental Digital Content, https://links.lww.com/MD/R372. Two researchers conducted the evaluation (WYK and CZM). If the results were inconsistent and divergent, the final results were determined after the third researcher (SJT) participated in the discussion. Following the discussion, the 3 researchers concluded and documented the consistent results in RevMan 5.3 to create a quality evaluation chart.

### 2.6. Statistical analysis

Heterogeneity in the literature was analyzed using Meta Dics 1.4 software and *I*^2^. The literature shows no heterogeneity if the *I*^2^ value is <25%. The heterogeneity is considered small if the *I*^2^ value is between 25% and 50%. If *I*^2^ is between 50 and 75%, it indicates some heterogeneity. If *I*^2^ is >75%, there is significant heterogeneity. If *I*^2^ exceeds 50%, the random effects model analyzes the data. If *I*^2^ < 50%, the fixed effects model was used to analyze the data.^[4]^ Rvman5.3 and Meta Dics1.4 software were also used. The SEN, SPE, LR+, LR−, and AUC comparison values were calculated for risk prediction models.

### 2.7. Evidence synthesis

**Figure 1.**
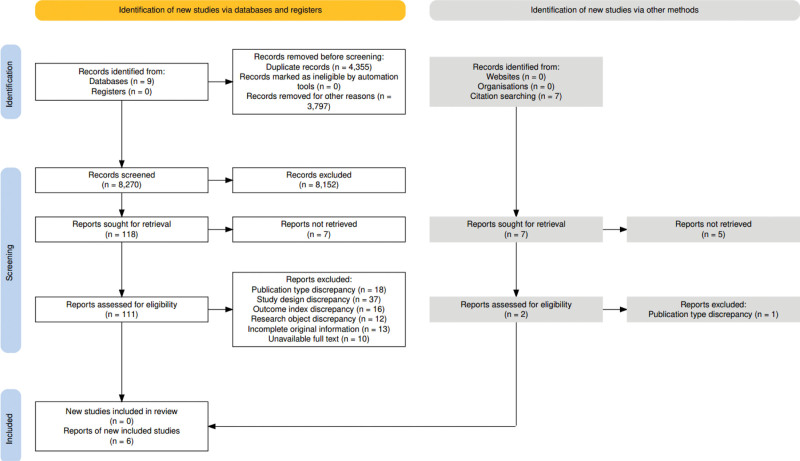
Literature selection framework. This is a flowchart depicting the study selection process, including the number of identified, screened, excluded, and included studies.

A total of 8277 articles were found (8270 in database search and 7 in manual search). After removing duplicates with EndNote 20, 3922 articles remained. After the initial screening, 125 papers were collected. Following additional screening, 6 pieces of literature were eventually included. Figure 1 provides the details of the literature screening process and results.

Two of the 6 included studies were prospective, while 4 were retrospective. In total, 24,911 ACS patients were enrolled. There were 18,443 (74%) male and 6468 (26%) female patients. The patients’ average age was 61 years, and the total number of in-hospital deaths was 1637, with 4 risk prediction models involved. Table 1 shows the details.

**Table 1:** The basic characteristics of the included studies.

Study	Year	Country	Type of research	Prediction model	Sample size	ST/NST	Age	Male/female	Number of deaths	Critical value
Ji et al^[15]^	2013	China	Retrospective	GRACE	390	278/112	63 ± 12	258/132	54	NR
				APACHEII						NR
				REMS						NR
Correia et al^[16]^	2010	Brazil	Retrospective	GRACE	154	84/70	71 ± 13	68/86	12	>136
				TIMI						>4
Gao et al^[17]^	2018	China	Prospective	GRACE	17,886	17,886/0	61.9 ± 12.4	13,685/4201	1153	≥155
				TIMI						>7
Ji et al^[18]^	2015	China	Retrospective	GRACE	394	394/0	65 ± 14	297/97	49	>185
				REMS						>5
				REMS2						>19
Wu et al^[19]^	2019	China	Prospective	GRACE	5896	0/5896	65.4 ± 12.1	4020/1876	353	≥141
				TIMI						>4
Chen et al^[20]^	2014	China	Retrospective	GRACE	191	0/191	69.7 ± 9.7 65.3 ± 8.9	115/76	16	>139
				TIMI						>3.5

APACHEII = acute physiology and chronic health evaluation II, GRACE = global registry of acute coronary events, NR = no record, NST = non-ST-segment elevation myocardial infarction, REMS = rapid emergency medicine score, ST = ST-segment elevation myocardial infarction, TIMI = thrombolysis in myocardial infarction.

### 2.8. Bias risk assessment of included studies

In this study, the PROBAST evaluation tool was used to assess the risk of bias in the articles in 4 areas: participants, predictors, results, and analysis. As shown in the table, there was a low risk of literature bias. Four articles posed an “unknown” bias risk. Overall, the literature did not pose a “high” risk of bias (Table 2).

**Table 2:** Risk of bias assessment for the included studies.

Study	Bias risk				Applicability			Total	
	Participant	Predictor	Result	Data analysis	Participant	Predictor	Result	Bias risk	Use
Ji et al^[15]^	+	?	−	?	+	+	−	−	−
Correia et al^[16]^	+	+	+	+	−	−	−	+	−
Gao et al^[17]^	+	+	+	+	−	−	−	+	−
Ji et al^[18]^	?	+	+	?	−	−	−	?	−
Wu et al^[19]^	+	+	+	+	−	−	−	+	−
Chen et al^[20]^	?	+	+	?	−	−	−	?	−

+: low concern for low offset risk/suitability; −: high concern for high offset risk/applicability.

?: unknown migration risk/unknown suitability concern.

### 2.9. Evaluation of the comparative accuracy of risk prediction models

#### 2.9.1. Comparison of accuracy between GRACE and TIMI

**Figure 2.**
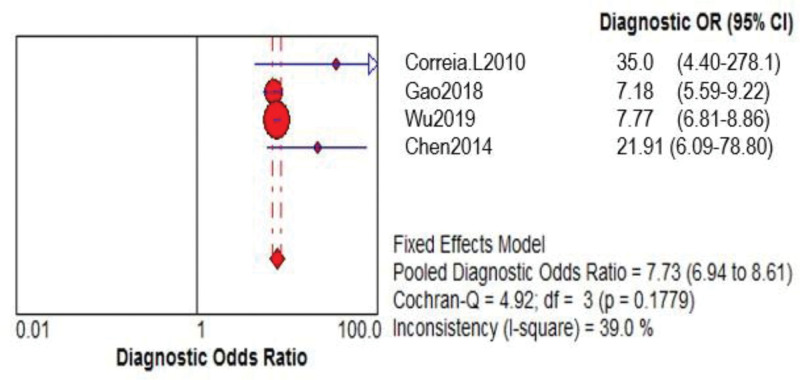
GRACE model DOR merge forest plot. CI = confidence interval, DOR = diagnostic odds ratio, GRACE = global registry of acute coronary events, OR = odds ratio.

**Figure 3.**
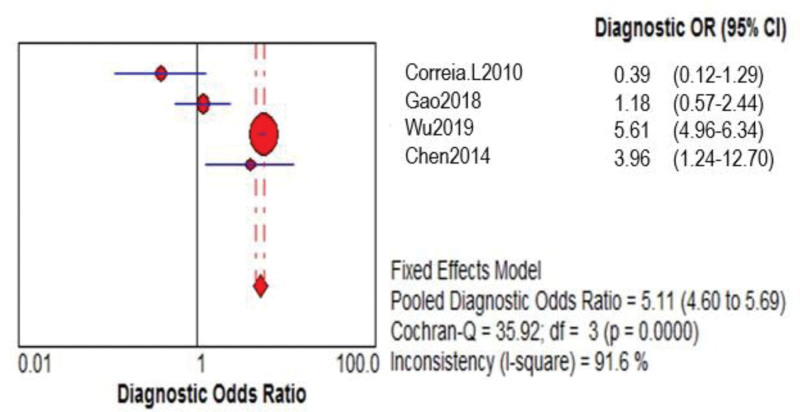
TIMI model DOR merge forest plot. CI = confidence interval, DOR = diagnostic odds ratio, OR = odds ratio, TIMI = thrombolysis in myocardial infarction.

**Figure 4.**
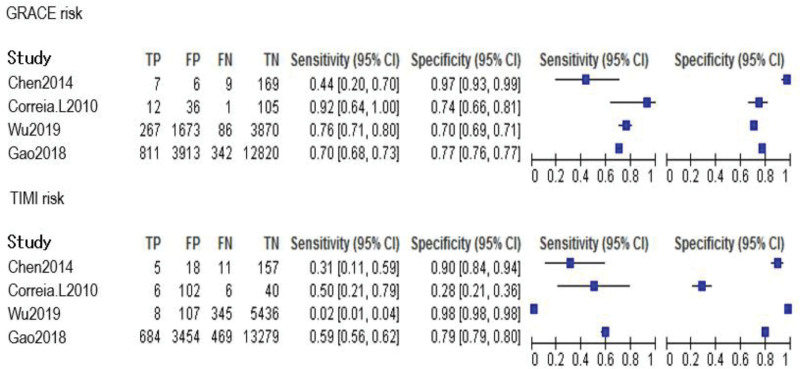
Forest plot of accuracy comparison between GRACE and TIMI. CI = confidence interval, GRACE = global registry of acute coronary events, TIMI = thrombolysis in myocardial infarction.

**Figure 5.**
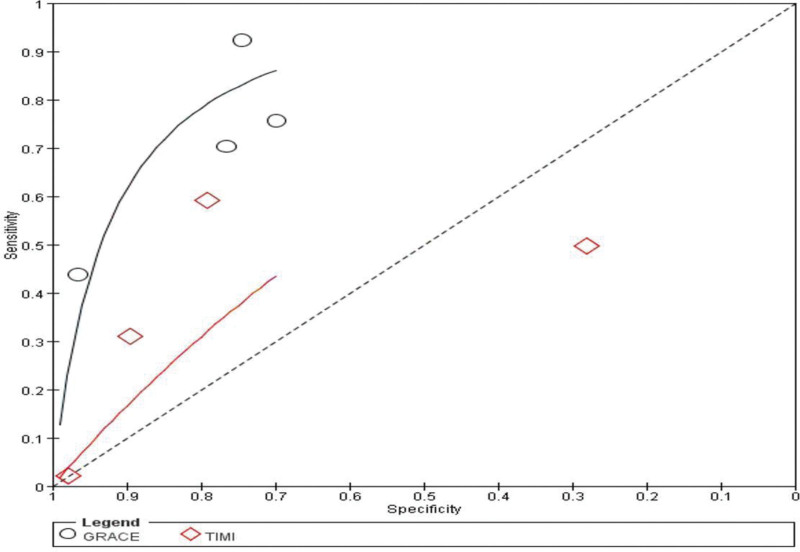
SROC curve comparison between the GRACE and the TIMI. GRACE = global registry of acute coronary events, SROC = summary receiver operating characteristic, TIMI = thrombolysis in myocardial infarction.

Four of the 6 papers included a comparison of the GRACE and TIMI risk prediction models. Two of these studies were prospective, while 2 were retrospective. A total of 24,127 ACS patients were enrolled. The GRACE risk prediction model had a higher SEN than the TIMI risk prediction model (0.71 > 0.46), as did the TIMI risk prediction model’s specificity (SPE; 0.84 > 0.75; Figs. 2–4 provides the detailed data analysis). The GRACE risk prediction model had significantly higher AUC value compared to the TIMI risk prediction model (0.79 > 0.59), as did the discrimination (Fig. 5). Table 3 provides a detailed comparison of the accuracy of the GRACE and TIMI risk prediction models.

**Table 3:** Accuracy comparison between the GRACE and the TIMI.

Prediction model	SEN (95% CI)	SPE (95% CI)	LR+ (95% CI)	LR− (95% CI)	DOR (95% CI)	AUC
GRACE	0.71 (0.69-0.74)	0.75 (0.75-0.76)	2.89 (2.78-3.00)	0.38 (0.35-0.41)	7.73 (6.94-8.61)	0.79
TIMI	0.46 (0.43-0.48)	0.84 (0.83-0.84)	1.64 (0.75-3.61)	0.91 (0.25-3.31)	5.11 (4.60-5.69)	0.59

AUC = area under the curve, CI = confidence interval, DOR = diagnostic odds ratio, GRACE = global registry of acute coronary events, LR+ = positive likelihood ratio, LR− = negative likelihood ratio, SEN = sensitivity, SPE = specificity, TIMI = thrombolysis in myocardial infarction.

#### 2.9.2. Comparison of accuracy between GRACE and REMS

**Figure 6.**
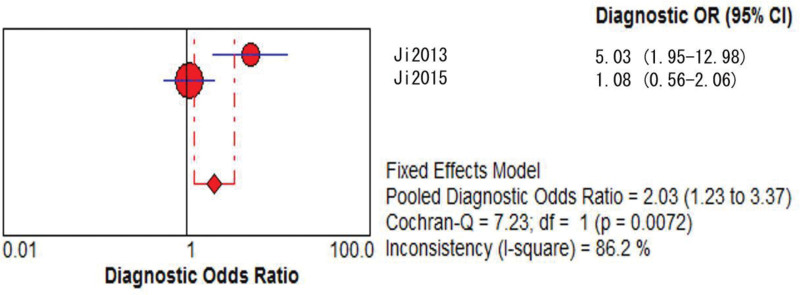
GRACE model DOR merge forest plot. CI = confidence interval, DOR = diagnostic odds ratio, GRACE = global registry of acute coronary events, OR = odds ratio.

**Figure 7.**
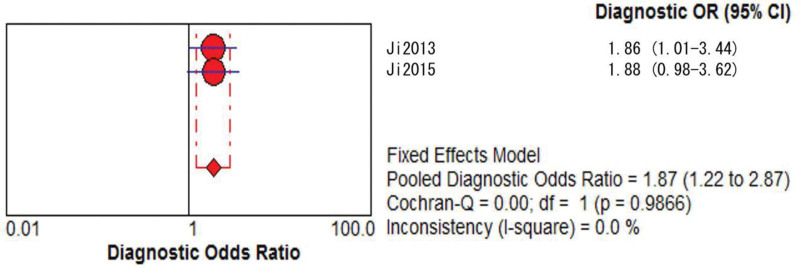
REMS model DOR merge forest plot. CI = confidence interval, DOR = diagnostic odds ratio, OR = odds ratio, REMS = rapid emergency medicine score.

**Figure 8.**
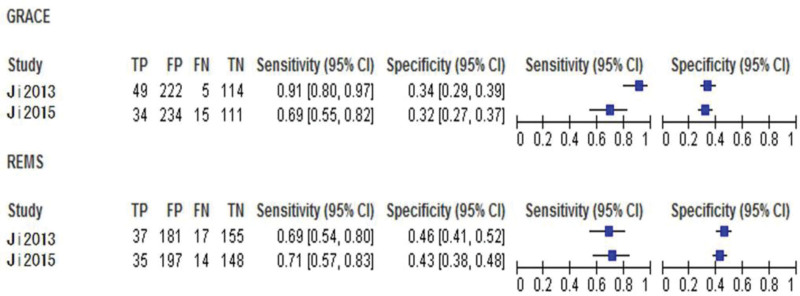
Forest plot of accuracy comparison between the GRACE and the REMS. CI = confidence interval, GRACE = global registry of acute coronary events, REMS = rapid emergency medicine score.

**Figure 9.**
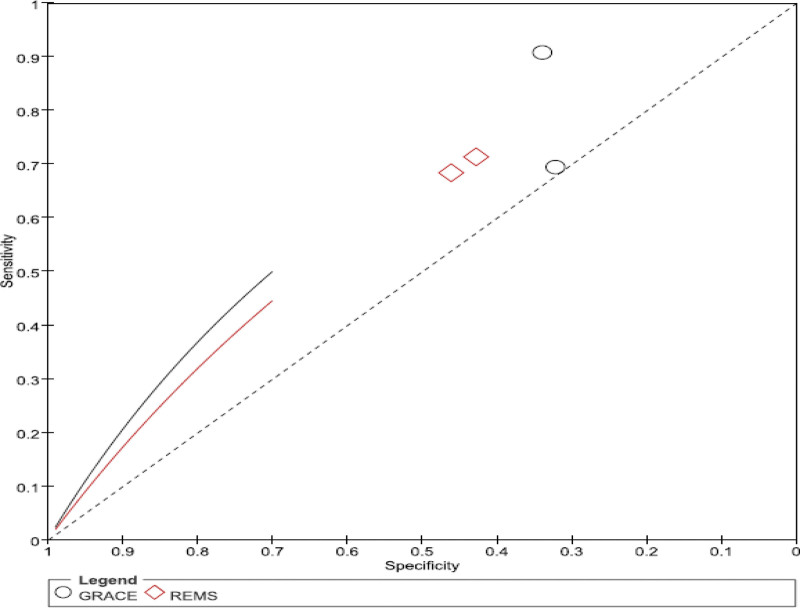
SROC curve comparison between the GRACE and the REMS. GRACE = global registry of acute coronary events, REMS = rapid emergency medicine score, SROC = summary receiver operating characteristic.

Two of the 6 articles included a comparison of the GRACE and REMS risk prediction models for the risk of death in ACS patients. Both studies were retrospective. A total of 784 ACS patients were enrolled. The calibration revealed that the REMS risk prediction model had a higher SEN than the GRACE risk prediction model (0.78 > 0.71), as well as a higher SPE than the REMS risk prediction model (0.75 > 0.46). Therefore, while the GRACE risk prediction model and the REMS risk prediction model have the same level of calibration, the AUC value of the GRACE risk prediction model is significantly higher than that of the REMS risk prediction model (0.73 > 0.41), indicating that the GRACE risk prediction model has a higher degree of discrimination (Figs. 6–9) for detailed data analysis. Therefore, the GRACE risk prediction model outperforms the REMS risk prediction model. Table 4 provides a detailed data comparison.

**Table 4:** Accuracy comparison between the GRACE and the REMS.

Prediction model	SEN (95% CI)	SPE (95% CI)	LR+ (95% CI)	LR− (95% CI)	DOR (95% CI)	AUC
GRACE	0.71 (0.69–0.74)	0.75 (0.74–0.76)	1.62 (0.82–3.22)	0.48 (0.25–0.95)	2.03 (1.23–3.37)	0.73
REMS	0.78 (0.71–0.85)	0.46 (0.43–0.49)	1.45 (1.05–2.00)	0.44 (0.19–1.02)	1.87 (1.22–2.87)	0.41

AUC = area under the curve, CI = confidence interval, DOR = diagnostic odds ratio, GRACE = global registry of acute coronary events, LR+ = positive likelihood ratio, LR− = negative likelihood ratio, REMS = rapid emergency medicine score, SEN = sensitivity, SPE = specificity.

#### 2.9.3. Accuracy comparison of GRACE, APACHE II, and REMS

A retrospective literature review compared the accuracy of 3 risk prediction models: the GRACE, REMS, and APACHE II models. The study included 390 patients (258 males and 132 females). The study used the descriptive method because combining data from a single document was impossible. The calibration of the research data results revealed that the SEN of the APACHE II risk prediction model was higher than that of the REMS risk prediction model (0.75 > 0.68). In contrast, the SPE of the REMS risk prediction model was higher than that of the APACHE II risk prediction model (0.46 > 0.22). Therefore, the APACHE II risk prediction model has the same calibration degree as the REMS risk prediction model, but its AUC value is significantly higher (0.82 > 0.61). It demonstrates that the discrimination of the APACHE II model is better than the REMS model.

The GRACE model is better calibrated than the APACHE II model (SEN 0.90 > 0.75, SPE 0.33 > 0.22) and has a higher distinction than the APACHE II model (0.86 > 0.82), implying that it outperforms the latter.

After comprehensive consideration, the GRACE risk prediction model outperforms the APACHE II and REMS models. Table 5 shows the detailed data.

**Table 5:** Accuracy comparison of the GRACE, the APACHE II and the REMS.

Prediction model	SEN (95% CI)	SPE (95% CI)	LR+ (95% CI)	LR− (95% CI)	AUC
REMS	0.68 (0.54–0.80)	0.46 (0.40–0.51)	1.27 (1.03–1.56)	0.68 (0.45–1.01)	0.61
APACHE II	0.75 (0.62–0.86)	0.22 (0.18–0.27)	0.97 (0.83–1.14)	1.07 (0.65–1.76)	0.82
GRACE	0.90 (0.78–0.96)	0.33 (0.28–0.39)	1.37 (1.22–1.54)	0.27 (0.11–0.63)	0.86

APACHEII = acute physiology and chronic health evaluation II, AUC = area under the curve, CI = confidence interval, GRACE = global registry of acute coronary events, LR+ = positive likelihood ratio, LR− = negative likelihood ratio, REMS = rapid emergency medicine score, SEN = sensitivity, SPE = specificity.

## 3. Discussion

### 3.1. Accuracy comparison between GRACE and TIMI

The data from this study indicates that the GRACE risk prediction model is the best. One study^[21]^ suggested that the difference between the 2 risk prediction models is because the GRACE risk prediction model is a large-scale, multicenter, international clinical study with a relatively comprehensive population. The GRACE risk prediction model contains predictive factors, such as blood pressure, cardiac function, hemodynamic variables, and heart rate, that have been confirmed to be independent risk factors for prognosis.^[22,23]^ Additionally, the GRACE risk prediction model places a high value on the influence of renal dysfunction as a predictor of the prognosis of ACS patient. Renal dysfunction has also been shown to be a close risk factor related to the prognosis of ACS patients, which is an independent risk factor for the risk of in-hospital mortality.^[22,24]^ Renal dysfunction is primarily common in elderly female patients. Therefore, the GRACE risk prediction model is more complete and accurate when assessing female patients. It explains why the TIMI risk prediction model has a lower accuracy than the GRACE risk prediction model. This study emphasizes the importance of using a risk prediction model to complement clinical assessment. In the individualized risk assessment of ACS patients, the clinical staff’s professional abilities must be combined to develop better individual treatment strategies.^[25]^

However, in clinical practice, the GRACE risk prediction model is used less frequently than the TIMI risk prediction model owing to its greater complexity. Lyon et al^[26]^ pointed out that the GRACE risk prediction model and the TIMI risk prediction model had similar prediction accuracy for emergency chest pain patients because their disease conditions were simpler than those of hospitalized patients. The TIMI risk prediction model has some advantages over the GRACE risk prediction model because it is simpler to use. However, the GRACE risk prediction model’s superior accuracy over the TIMI risk prediction model makes up for its complexity. The GRACE risk prediction model has a significant advantage because of the strong correlation between the included risk factors and the prognosis of ACS patients, such as heart rate, plasma creatinine, and Killip classification. However, variables from the TIMI risk prediction model, such as angina within 24 hours, aspirin use, and the degree of coronary artery occlusion, were less related to the outcome events. It also contributes to the accuracy of the TIMI risk prediction model. This is consistent with the findings of Goncalves,^[27]^ who demonstrated that the GRACE risk prediction model outperformed the TIMI risk prediction model, with a statistically significant difference in the C statistic (AUC) between the 2 models. Correia et al^[16]^ concluded that the GRACE risk prediction model had good prognosis prediction ability for ACS patients. Therefore, it was proposed that the GRACE risk prediction model be used in conjunction with the TIMI risk prediction model to better predict the mortality risk of hospitalized ACS patients.

The results of this study showed that the GRACE risk prediction model has a significantly higher pooled effect size than the TIMI risk prediction model. This is consistent with previous studies,^[4,25,28–30]^ which found that the GRACE risk prediction model outperformed the TIMI risk prediction model in predicting mortality risk in ACS patients. By comparing the 2 risk prediction models, this study suggests that the nursing staff use the GRACE risk prediction model to assess ACS patients.

#### 3.1.1. Comparison of accuracy between GRACE and REMS

Ji et al^[15]^ conducted a study in 2013 with 390 AMI patients, using a risk prediction model to assess the mortality risk of AMI patients within 30 days of onset. The research findings revealed that the GRACE risk prediction model had significant accuracy advantages over the REMS risk prediction model. In 2015, Ji et al^[18]^ improved the REMS risk prediction model by including 9 clinical indicators closely related to ST-segment acute coronary syndrome (ST-ACS) patients. The selected parameters were obtained more quickly and were not limited by equipment, so the evaluation time was shorter than that of the GRACE risk prediction model, significantly increasing REMS2’s evaluation efficiency and accuracy. The results indicated that the REMS2 risk prediction model outperformed the GRACE risk prediction model (AUC = 0.81 vs AUC = 0.73). However, this study’s average visit time of ST-ACS patients was 23 hours, significantly longer than the optimal treatment time of 4 hours, resulting in a long delay between disease onset and hospital visit, missing the optimal treatment time, and increasing the mortality of ST-ACS patients. Therefore, clinical multicenter research trials are still required to validate the accuracy of the REMS2 risk prediction model.

In a comprehensive comparison, the GRACE risk prediction model outperforms the REMS risk prediction model regarding pooled effect size. Therefore, we concluded that the GRACE risk prediction model outperformed the REMS risk prediction model in predicting mortality of ACS patients. By comparing the 2 risk prediction models, our study suggests that the nursing staff use the GRACE risk prediction model to assess ACS patients.

#### 3.1.2. Comparison of the accuracy of GRACE, APACHE II, and REMS

In 2013, Ji et al^[15]^ demonstrated that the GRACE model outperformed the APACHE II and REMS models in predicting the risk of death in ACS patients. Both the GRACE risk prediction model and the APACHE II risk prediction model exhibit excellent prediction ability, with the GRACE risk prediction model coming very close to the APACHE II risk prediction model in terms of the degree of discrimination. This is primarily due to the fact that the APACHE II risk prediction model considers the cardiovascular system and other systems such as the respiratory, digestive, endocrine, nervous, and immune systems.^[31,32]^ Its comprehensive evaluation of patients improves its ability to predict the risk of death. The APACHE II risk prediction model also has a good prediction effect on the evaluation of critical diseases of various systems, and it is the world’s most widely used and authoritative risk prediction model for evaluating the severity of illness and prognosis of critically ill patients.^[33,34]^ However, the APACHE II risk prediction model has more parameters and is more complex to operate, increasing the workload of the medical and nursing staff in clinical practice. The finding of this study suggested that the GRACE risk prediction model is better suited for assessing the prognosis of ACS patients. It is recommended that nursing staff use the GRACE risk prediction model to evaluate ACS patients.

In recent years, machine learning prediction models have demonstrated remarkable potential by simultaneously processing multidimensional, complex, and large-scale patient cases and laboratory data, thereby addressing the limitations of single-factor analysis in screening predictive factors. Study^[35]^ indicates that while machine learning prediction models perform effectively, they lack interpretability. This may be attributed to the nonuniform standardization of patient clinical data collection and the prevalence of missing data during analysis. Moreover, although processing large and complex datasets is well-suited for laboratory analysis and computation, its application and promotion in clinical settings remain challenging due to resource and operational constraints. Given the limited number of machine learning models examined in this study and the absence of comparative studies, therefore, machine learning-based risk models were excluded from this study. Nevertheless, the integration of machine learning and conventional models within future meta-analyses or hybrid evaluation frameworks to guide personalized clinical decision-making remains a topic worthy of further exploration.

## 4. Limitations of the study

The literature included in this study comprises numerous retrospective studies, with the most critical limitation being the very small number of studies incorporated in each comparison, which to some extent increased the risk of bias in the results. Among the included studies, 5 were conducted in China and 1 in Brazil, indicating a potential country bias. As China has a large population and carries a significant cardiovascular disease burden, accurate risk scoring is crucial for cost-effective primary prevention. This necessity has prompted numerous studies of various prediction models in China. In future research, we aim to further expand the countries and populations, as well as increase the diversity of risk models included, to enhance the scientific rigor and generalizability of the studies. Furthermore, the evaluation index for the discrimination of the prediction model was relatively broad and had some limitations, which limit recommendations for research decisions and risk prediction models. PROBAST assessments revealed that several studies exhibited an “unclear” risk of bias in the data analysis domain, often due to incomplete reporting of modeling and validation methods. The use of inappropriate statistical approaches or the omission of key methodological principles may increase the risk of bias. Future studies should closely follow PROBAST guidelines and provide detailed descriptions of modeling procedures, validation processes, and associated datasets to enhance applicability and reduce bias in prediction models for mortality in ACS.

## 5. Conclusions

Available data from a limited number of studies suggest that the GRACE risk prediction model is highly accurate in assessing the clinical risk of death in patients with ACS. The evidence indicates that GRACE is superior to the TIMI, APACHE II, and REMS models, though the final choice may also depend on clinical context and practicality, such as TIMI’s simplicity. Furthermore, while this study promotes the use of the GRACE model to determine mortality risk in ACS patients – aiming to improve the quality of care and effectively reduce mortality – it should be acknowledged that the applicability of these models may vary based on local healthcare settings, patient populations, and available resources.

## Acknowledgments

The authors wish to thank all authors whose published articles were included in their meta-analysis.

## Author contributions

**Conceptualization:** Yike Wang, Jiantong Shen.

**Data curation:** Yike Wang, Zhimei Chen, Jianping Song.

**Formal analysis:** Jianping Song.

**Funding acquisition:** Yike Wang, Zhimei Chen.

**Investigation:** Meijuan Lan.

**Methodology:** Meijuan Lan.

**Project administration:** Jiantong Shen.

**Writing – original draft:** Yike Wang, Jiantong Shen.

**Writing – review & editing:** Yike Wang, Zhimei Chen.

## Supplementary Material


